# Single molecule network analysis identifies structural changes to caveolae and scaffolds due to mutation of the caveolin-1 scaffolding domain

**DOI:** 10.1038/s41598-021-86770-6

**Published:** 2021-04-08

**Authors:** Timothy H. Wong, Ismail M. Khater, Bharat Joshi, Mona Shahsavari, Ghassan Hamarneh, Ivan R. Nabi

**Affiliations:** 1 Life Sciences Institute, Department of Cellular and Physiological Sciences, University of British Columbia, Vancouver, BC V6T 1Z3 Canada; 2 School of Computing Science, Simon Fraser University, Burnaby, BC V5A 1S6 Canada; 3 School of Biomedical Engineering, University of British Columbia, Vancouver, BC V6T 1Z3 Canada

**Keywords:** Structural biology, Organelles, Focal adhesion, Super-resolution microscopy, Cancer, Computational science

## Abstract

Caveolin-1 (CAV1), the caveolae coat protein, also associates with non-caveolar scaffold domains. Single molecule localization microscopy (SMLM) network analysis distinguishes caveolae and three scaffold domains, hemispherical S2 scaffolds and smaller S1B and S1A scaffolds. The caveolin scaffolding domain (CSD) is a highly conserved hydrophobic region that mediates interaction of CAV1 with multiple effector molecules. F92A/V94A mutation disrupts CSD function, however the structural impact of CSD mutation on caveolae or scaffolds remains unknown. Here, SMLM network analysis quantitatively shows that expression of the CAV1 CSD F92A/V94A mutant in CRISPR/Cas CAV1 knockout MDA-MB-231 breast cancer cells reduces the size and volume and enhances the elongation of caveolae and scaffold domains, with more pronounced effects on S2 and S1B scaffolds. Convex hull analysis of the outer surface of the CAV1 point clouds confirms the size reduction of CSD mutant CAV1 blobs and shows that CSD mutation reduces volume variation amongst S2 and S1B CAV1 blobs at increasing shrink values, that may reflect retraction of the CAV1 N-terminus towards the membrane, potentially preventing accessibility of the CSD. Detection of point mutation-induced changes to CAV1 domains highlights the utility of SMLM network analysis for mesoscale structural analysis of oligomers in their native environment.

## Introduction

Gene mutations resulting in single amino acid changes have functional impact on protein activity leading to disease. Proteins form macromolecular complexes in the mesoscale range (10–200 nm) in the cell yet how mutations alter the structure of these complexes remains difficult to define. X-Ray crystallography and NMR structural analysis can determine the impact of mutations on protein structure at the atomic level^1,2^. Biochemical approaches, including elegant proximity-based proteomic approaches can determine the impact of protein mutations on protein–protein interactions^3^. CryoEM provides structural analysis of macromolecular complexes but can only with difficulty localize specific protein components within the complex^4^. How functional mutations impact distribution of the mutated protein within mesoscale macromolecular structures remains a challenge. Single molecule localization microscopy (SMLM) is a fluorescence based super-resolution microscopy approach that can localize proteins with ~ 15–20 nm X–Y (lateral) resolution and, for astigmatic lens 3D SMLM, ~ 25–50 nm Z (axial) resolution^5,6^. Application of network/graph analysis and machine learning to SMLM point distributions has allowed us to develop a computational pipeline to determine the 3D molecular architecture of macromolecular complexes^7–10^. Here we show that SMLM network analysis can detect structural changes to ~ 100 nm diameter caveolae and smaller non-caveolar scaffold domains due to point mutations of the highly conserved caveolin scaffolding domain (CSD).

Caveolin-1 (CAV1) is a 178 amino acid integral membrane protein essential for the biogenesis of caveolae, 50–100 nm plasma membrane invaginations consisting of 140–150 CAV1 molecules and whose formation requires the adaptor protein CAVIN1, also known as PTRF^1,11–14^. In the absence of CAVIN1, CAV1 forms non-caveolar scaffold domains^15^; as both caveolae and scaffolds are smaller than the diffraction limit (~ 250 nm) resolving these structures by fluorescent microscopy requires the use of super-resolution microscopy approaches. SMLM has been used to image CAV1 with around 20 nm resolution^10,16,17^. In our previous studies, application of 3D SMLM network analysis was used to segment and identify caveolae and scaffolds from CAV1 clusters and obtain 3D structural information^8,10^. SMLM network analysis showed that caveolae are composed of a modular CAV1 coat which corresponds to structures determined by cryoelectron microscopy^8,18,19^. It also identified distinct scaffolds: small S1A scaffolds corresponding to SDS-resistant CAV1 scaffolds described above; S1B scaffolds that correspond to S1A dimers and larger hemispherical S2 scaffolds^8,10^. Interestingly, CAVIN1 was shown to associate with both caveolae and scaffolds^7,9^.

The CAV1 CSD is a highly conserved hydrophobic region from amino acids 82–101^20,21^. The CSD mediates CAV1 interaction with various proteins including Src family tyrosine kinases, eNOS and focal adhesion proteins^1,21–27^. A F92A point mutation in the essential F92TVT95 segment of the CSD was sufficient to inhibit CAV1-eNOS interaction and prevent the ability of CAV1 to inhibit nitric oxide production^23,28,29^. Further supporting the role of CSD activity, the addition of cavtratin, a cell-permeable peptide containing the CSD amino acids (82–101) of CAV1 inhibits eNOS and blocks nitric oxide release in vitro^30,31^. A double point mutation in the CSD (F92A/V94A) abolishes CAV1 co-immunoprecipitation with the EphB1 receptor after ephrin ligand stimulation, therefore suggesting that interactions between EphB1 receptor tyrosine kinase and CAV1 depend on the CSD^32^. Furthermore, the F92A/V94A CSD mutant prevents CAV1 CSD interactions with insulin receptor, insulin receptor kinase activity and downstream Elk-1 and Erk-2 phosphorylation^25^. FRAP of the insulin receptor shows that expression of wild-type CAV1, but not the F92A/V94A CSD mutant, immobilizes insulin receptor^33^. Similarly, CAV1 reduction of the cell surface diffusion of both GM1-bound cholera toxin b subunit and EGFR, as well as EGFR signalling, is prevented by the F92A/V94A CSD CAV1 mutation^34^. Further, F92A/V94A CSD mutation and cavtratin inhibit pCAV1-dependent cancer cell migration and focal adhesion tension^26^; deletion of the CSD prevents inhibition of HeLa cell migration by CAV1 overexpression^35^.

However, in spite of the extensive data supporting functional interactions of the CSD and their disruption by F92A and V94A mutations, structural impact of these mutations on CAV1 domains is not known. The CSD is a hydrophobic domain thought to be either embedded in or in close proximity to the cell membrane, raising questions as to its accessibility to interacting proteins^27,36,37^. Conformational changes to CAV1 domain structures due to Y14 phosphorylation increased CSD interaction with focal adhesion proteins, consistent with reported Y14 phosphorylation-induced CAV1 conformational changes that may increase CSD accessibility^26,38,39^. Expression of wild-type CAV1 or the F92A CAV1 mutant in endothelial cells showed that while the F92A mutant increased nitric oxide bioavailability in vivo, it fractionated similarly to wild-type CAV1 on sucrose gradients and similar numbers of caveolae per length of plasma membrane were observed by electron microscopy^40^. However, cryoelectron tomography showed that, while present at the plasma membrane, upon truncation and removal of the entire CSD CAV1 did not form caveolae^41^. Here, we applied SMLM network analysis to identify structural changes induced to caveolae and scaffolds by the F92A/V94A mutation of the CAV1 CSD. To analyze the CSD mutant in a CAV1 null background, we knocked out CAV1 using CRISPR/Cas in a MDA-MB-231 breast cancer cell clone. We show that the CAV1 CSD point mutation reduces the size and increases the elongation of caveolae and scaffolds. In particular, impact of CSD F92A/V94A mutation on the size and shape of intermediate S2 and S1B scaffolds is more pronounced, supporting a role for these scaffold domains in CSD activity. Importantly, this study demonstrates that SMLM network analysis can detect structural changes to protein oligomers induced by point mutations.

## Results

### CAV1 CRISPR/Cas9 knockout in MDA-MB-231 breast cancer cells

**Figure 1:**
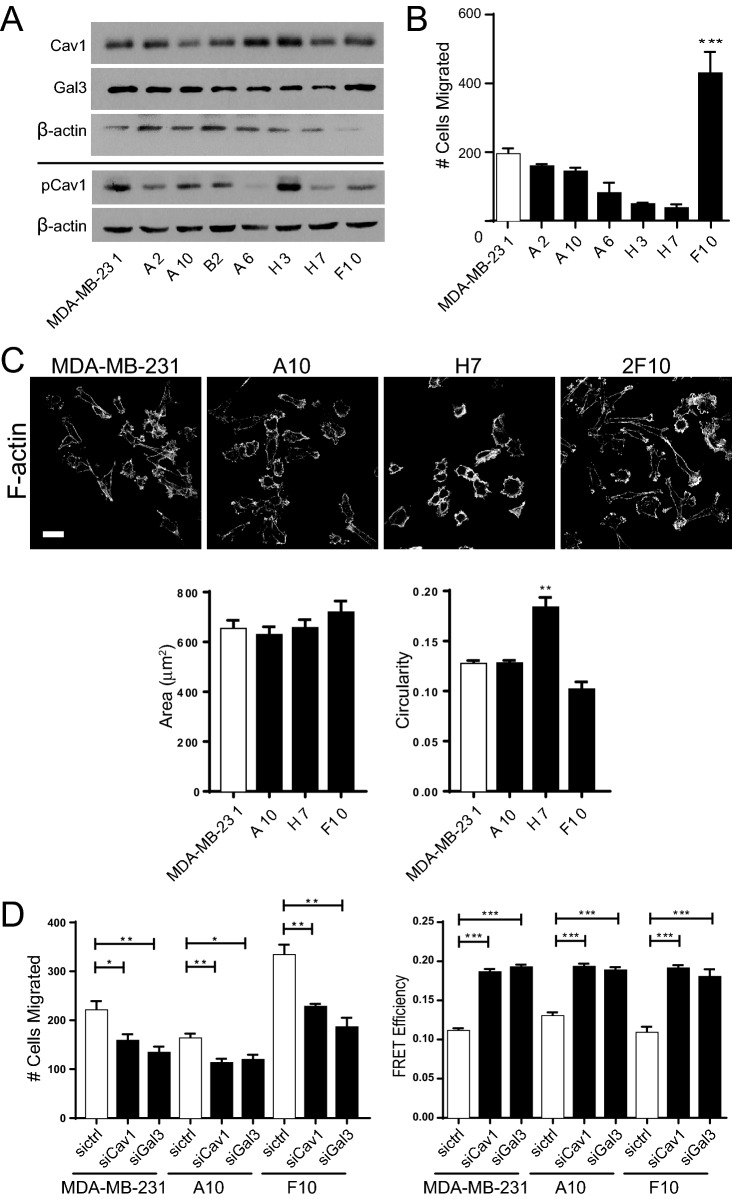
Generation of F10 sub-clone from parental MDA-MB-231. (**A**) Western blots of endogenous CAV1, Gal3 and β-actin and, separately, of pCAV1 and β-actin, in MDA-MB-231 sub-clones. (**B**) Quantification of the number of migrated cells in Transwell migration assays of sub clones and the parental MDA-MB-231 cells. Data represent mean ± SEM from at least three independent experiments (n > 3 for each clone). One-way analysis of variance (ANOVA) with Tukey post-test; ***p < 0.001. (**C**) Sub-clones fluorescently labelled with Alexa Fluor 488-Phallodin, showing differences in cell morphology. Scale bar, 30 µm. Cell area and circularity of F-actin labelled cells were quantified from three independent experiments (n = 3 with at least 40 cells quantified per sub-clone in each experiment; ANOVA with Tukey post-test; **p < 0.01). (**D**) Parental MDA-MB-231 and sub-clones A10 and F10 CAV1 and Gal3 siRNA knockdown. Bar graphs of mean ± SEM cells migrated in Transwell migration assays (n = 5; ANOVA with Tukey post-test; *p < 0.05; **p < 0.01). FRET efficiency quantified in focal adhesions of cells transfected with a vinculin FRET sensor (n = 3; ANOVA with Tukey post-test; ***p < 0.001).

In order to express the F92A/V94A CAV1 mutant in a CAV1-free environment, we generated a CAV1 knockout MDA-MB-231 cell line adapting a modified CRISPR/Cas9 strategy to mitigate heterogeneity observed in cancer cell lines^42^. Heterogeneity has been observed in other cancer cell lines across different laboratories and passage numbers; within single cell derived MCF7 clones, there were genetic changes associated with gene expression and differences in anti-cancer drug responses^43,44^. We cloned the parental MDA-MB-231 cell line by limiting dilution and evaluated the phenotype of single clones based on protein expression, cell morphology and cell migration and found substantial clonal heterogeneity. Cell lysates were probed for CAV1 as well as for Galectin-3 (Gal3) whose interaction with CAV1 controls CAV1 regulation of EGFR signalling, focal adhesion dynamics and cell migration^26,34,45–47^. Substantial variation in CAV1, pCAV1 and Gal3 levels were observed amongst the clones (Fig. 1A). Migratory ability of clones was the same or reduced relative to parental MDA-MB-231 cell line, except for the F10 clone that showed significantly increased migratory ability, that did not correlate with CAV1, Gal3 or pCAV1 levels (Fig. 1B). Confocal microscopy of phalloidin labeled F-actin showed that less migratory clones, such as H7, had circular and cuboidal shapes, while the more migratory F10 clone was elongated with actin-rich protrusions (Fig. 1C). Quantification of phalloidin labeled cells showed that A10, H7 and F10 sub-clones had a similar cell area but varied circularity, with H7 more circular and A10 and F10 similar to parental MDA-MB-231 cells (Fig. 1C). A10 showed reduced and F10 increased Transwell migration relative to parental MDA-MB-231 cells (Fig. 1B); siRNA knockdown of CAV1 and Gal3 reduced Transwell cell migration for parental MDA-MB-231 cells and the A10 and F10 clones (Fig. 1D, Supp. Fig. 1). In addition, siRNA knockdown of both CAV1 and Gal3 increased FRET efficiency of the vinculin FRET tension sensor^48^, indicative of reduced focal adhesion tension (Fig. 1D), as we previously reported for PC3 prostate cancer cells^26^. Regulation of vinculin tension in MDA-MB-231 breast cancer cells is therefore both CAV1- and Gal3-dependent.

**Figure 2:**
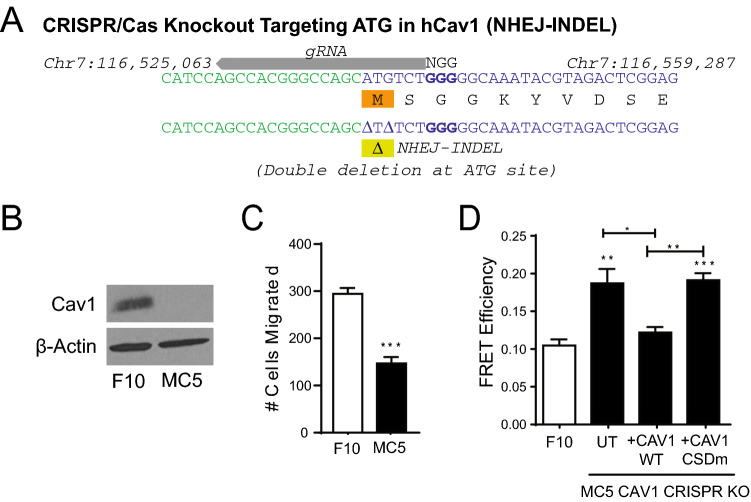
CAV1 CRISPR knockout from F10 sub-clone. (**A**) Schematic of the gRNA designed to target the ATG start site of the CAV1 sequence. (**B**) Western blot of CAV1 in MC5 CRISPR/CAS9 knockout and F10 sub-clone used to generate MC5. (**C**) Bar graphs of mean ± SEM of cells migrated between parental F10 and MC5 quantified from Transwell migration assays (n = 5; two-tailed unpaired t test; ***p < 0.001). (**D**) Vinculin FRET efficiency of F10 parental line and MC5 rescued with CAV1 wildtype and the CSD mutant (n = 3; ANOVA with Tukey post-test; *p < 0.05; **p < 0.01). See Supp. Fig. 5 for complete blots that were cropped for Fig. 1A,D.

We chose to use the F10 clone for further study as it has a similar mesenchymal morphology and increased migratory ability relative to that of MDA-MB-231 cells^49^ as well as high CAV1 expression and CAV1- and Gal3-dependent migration and focal adhesion tension. To knockout CAV1 using CRISPR/Cas9, we designed a guided RNA (gRNA) targeting the ATG start codon of human CAV1 (Fig. 2A). The CAV1 knockout clone MC5 expressed no detectable CAV1 protein and presented significantly reduced Transwell migration relative to the parental F10 clone and increased FRET efficiency of the vinculin tension sensor reflecting reduced vinculin tension; transient transfection of CAV1 wildtype, but not the CAV1 F92A/V94A CSD mutant reduced FRET efficiency of the vinculin tension sensor (Fig. 2B–D). Confocal microscopy images presented no association of myc-tagged CAV1 wildtype or the F92A/V94A CSD mutant with Golgi or endoplasmic reticulum labelling. Large puncta present in cells expressing the CSD mutant were not associated with Golgi or ER and not present in peripheral regions presenting plasma membrane CAV1 labeling (Supp. Fig. 2).

### Single molecule localization microscopy network analysis of the F92A/V94A CSD mutant

**Figure 3:**
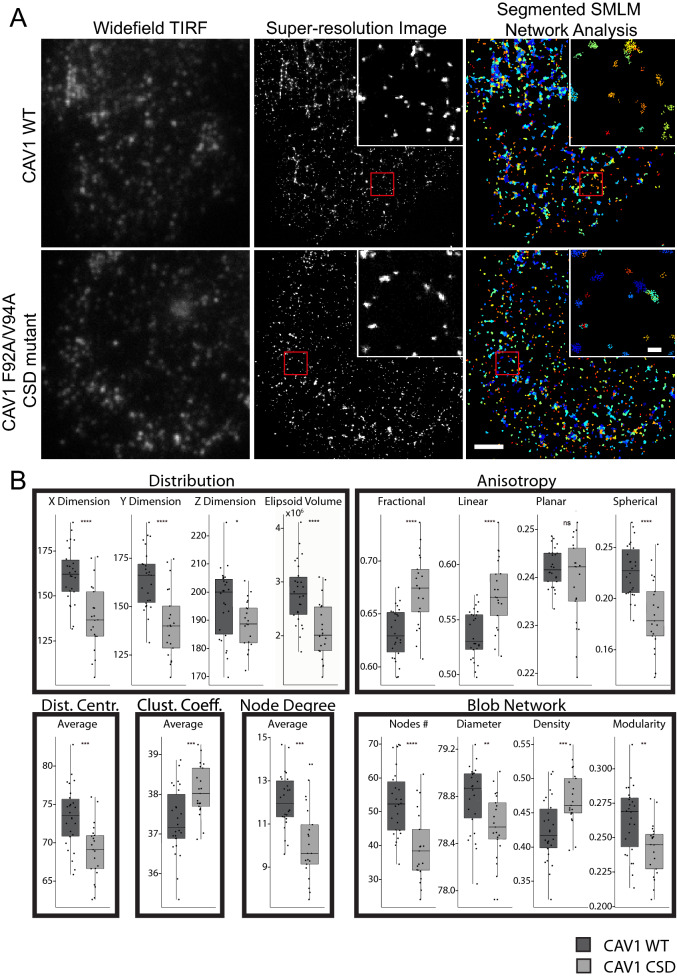
SMLM network analysis segments CAV1 clusters from SMLM data. (**A**) Representative TIRF wide-field imaging of CAV1 and SMLM GSD imaging of CAV1 in MC5 cells transfected with CAV1 WT or CSD mutant. 3D point clouds were processed with *3D SMLM Network Analysis*^10^ using iterative merging at 20 nm, filtering and segmentation to identify individual CAV1 blobs. Magnified SMLM and network analysis images of the boxed region highlights network analysis clusters (blobs) after processing. Scale bar, 2.5 µm; zooms, 250 nm. (**B**) Overall changes in features. Quantification of blob’s localizations distribution, anisotropy, localization’s distance to centroid, and blob’s network features between MC5 cells transfected with CAV1 WT or CSD mutant (n = 26 CAV1 WT cells, n = 21 CSD cells from four independent experiments; two-tailed unpaired t test; *p < 0.05; **p < 0.01; ***p < 0.001; ****p < 0.0001). The rest of the global CAV1 features are shown in Supp. Fig 3.

Super-resolution SMLM imaging was performed on the CAV1 knockout clone MC5 transiently transfected with myc-tagged CAV1 wildtype or the CSD F92A/V94A mutant and labeled with anti-CAV1 primary and Alexa647 conjugated secondary antibodies. The cells were imaged by TIRF widefield and 3D GSD-TRIF SMLM (Fig. 3A). The 3D point cloud of CAV1 wildtype and the CSD mutant transfected cells was generated by SMLM as an event list and processed by iterative merging, denoising filtration and segmentation into individual CAV1 blobs, as previously described (Fig. 3A)^10^. The merging threshold of 20 nm is set within the resolution limit of the system and generates caveolae with an average of 144 predicted CAV1 molecules^10,13^. 3D Network analysis quantifies a 28-descriptor vector (see Table S1 for description of all 28 features analyzed) from each blob that describes shape, topology, network and node features^8,10^. Quantification shows that there are significant differences globally between the features of all CAV1 wildtype and CSD mutant blobs. We observe a significant decrease in the X and Y dimensions and ellipsoid volume of the CSD mutant CAV1 blobs (Fig. 3B, Supp. Fig. 3). Absence of differences in Z height likely reflects the reduced axial resolution of 3D SMLM astigmatic lens SMLM^6^. Other notable differences are that CSD mutant blobs present fewer nodes (i.e., the reconstructed CAV1 localizations after applying the merging module), reduced node degree and reduced distance to centroid (Fig. 3B; see Supp. Fig. 3 for all features). CSD mutant blobs also present increased fractional and linear anisotropy and reduced spherical anisotropy (Fig. 3B). These data suggest that CSD mutation generally results in a reduced size and increased elongation of CAV1 blobs.

**Figure 4:**
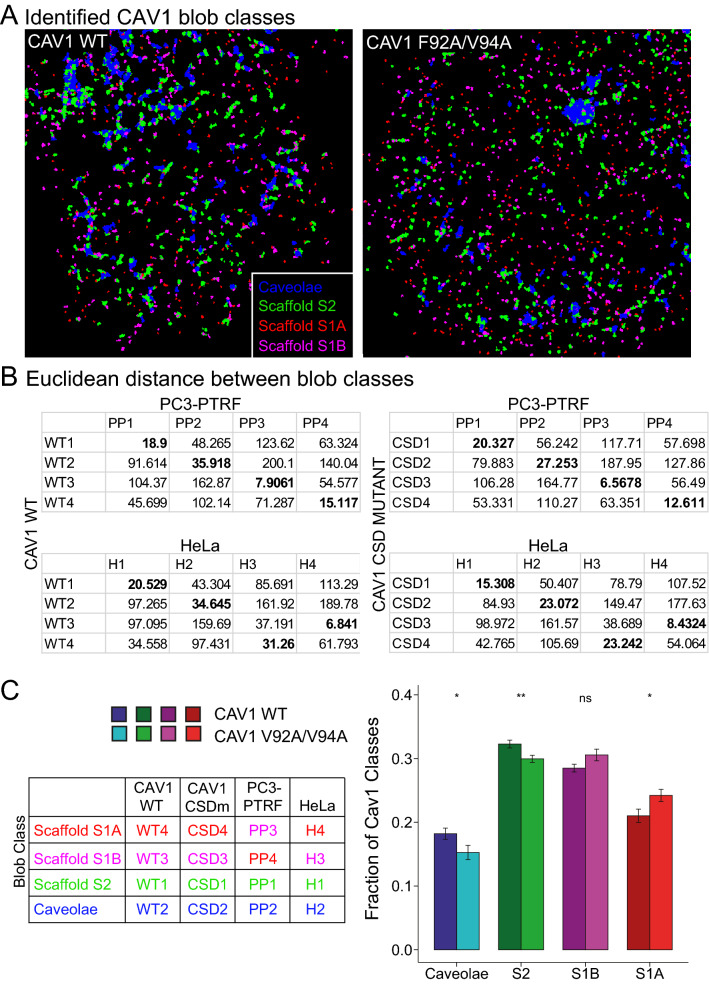
CAV1 domain class identification via similarity analysis using Euclidean distance, group matching, and fraction of CAV1 domains. (**A**) Representative images processed with blob identification by machine learning using *3D SMLM Network Analysis.* (**B**) Euclidean distance between blobs’ average features of the different classes of CAV1 WT (WT1-4) and CSD (CSD1-4) mutant to PTRF-expressing PC3 (PP1-4) and HeLa (H1-4) cells^8,10^. Bolded values correspond with (**C**) Identifying caveolae and S2, S1B and S1A scaffold blob groups from the shortest Euclidian distances. The classes are color coded based on the matched CAV1 domains across CAV1 WT, CSD, and the previously studied PC3-PTRF and HeLa SMLM data. Proportion of blob classes between CAV1 WT and CSD mutant (two-tailed unpaired t test; *p < 0.05; **p < 0.01).

To further understand the effects of the CSD mutant on specific CAV1 structures, we applied X-means unsupervised clustering to classify the blobs into groups and assess feature changes in each group. The clustering method found that for both CAV1 wildtype and CSD mutant, the optimal clustering was to split the blobs into four groups (Fig. 4A,B). Silhouette diagrams show efficiency of clustering of both CAV1 WT and CSD mutant blobs into 4 groups (Supp. Fig. 4). Matching these groups based on the similarity to CAV1 domains previously identified in HeLa and CAVIN1 expressing PC3 cells (PC3-PTRF) using the Euclidian distance of all 28 feature centres^8,10^ allowed us to designate the blobs as either caveolae, S2, S1B or S1A scaffolds (Fig. 4C). The CSD mutation results in a shift in the distribution of CAV1 structures from larger caveolae and hemispherical scaffolds with an increased abundance of smaller S1 scaffolds (Fig. 4C).

**Figure 5:**
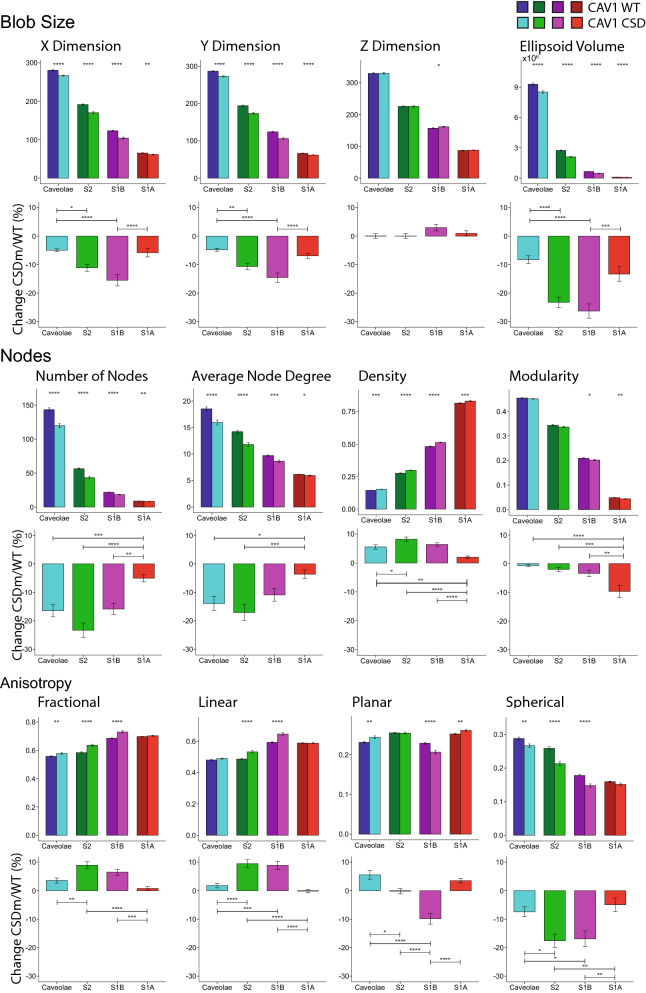
Changes in features of each class of CAV1 blobs. Bar graphs depicting changes in blob size, number of nodes, and anisotropy with respect to each class of blobs in CAV1 WT and CSD mutant (two-tailed unpaired t test; *p < 0.05; **p < 0.01; ***p < 0.001; ****p < 0.0001). Percent change of the features between CSD mutant and CAV1 WT (ANOVA with Tukey post-test; *p < 0.05; **p < 0.01; ***p < 0.001; ****p < 0.0001).

Quantification of the features for caveolae, S2, S1B and S1A between wild-type CAV1 and the CSD mutant shows that the CSD mutation decreases blob size, as reflected in smaller X and Y dimensions and volume of all CAV1 domains (Fig. 3B), with a more pronounced reduction in size for S1B and S2 scaffolds (Fig. 5). The reduced size of caveolae, S2 and S1B scaffolds is associated with increased network density, reduced number of nodes and reduced average node degree (Fig. 5). Modularity of caveolae and S2 scaffolds shows minimal changes suggesting that the overall structure of these larger CAV1 domains is retained (Fig. 5). In addition, CSD mutation is associated with shape changes to CAV1 blobs; the CSD mutation reduces spherical anisotropy for all classes of CAV1 blobs, and the effect that is more pronounced for S2 and S1B scaffolds than for caveolae and S1A scaffolds. Consistently, fractional and linear anisotropy are increased for CSD mutant S2 and S1B scaffolds. Planar anisotropy is decreased in S1B scaffolds and increased for caveolae (Fig. 5). Together, these data suggest that CSD mutation reduces the size and sphericity of caveolae and scaffolds inducing a more condensed, elongated form for complex CAV1 domains.

**Figure 6:**
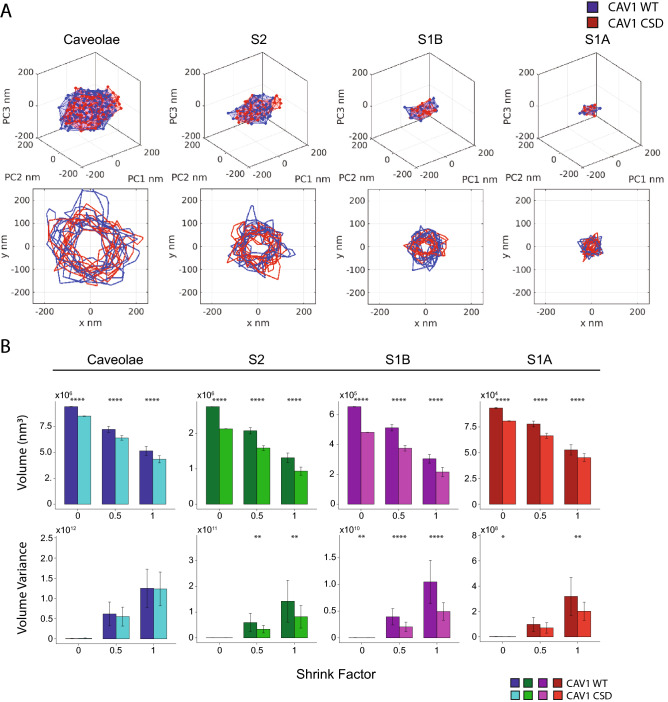
Convex hull analysis of CAV1 blobs. (**A**) 3D representation of the PCA for top blob from CAV1 WT and CSD based on the average of 28 network analysis features for the four CAV1 blob classes. Overlay of the 2D X–Y representation from the top 10 blobs closes to the average of CAV1 WT and CSD mutant features. (**B**) Bar graphs of the average blob volume and volume variance in the top 10 blobs of each cell at shrink factors of 0 (the most convex), 0.5, and 1 (the most indented) (two-tailed unpaired t test; *p < 0.05; ***p < 0.001; ****p < 0.0001).

To better characterize the shape changes associated with CSD mutation, we generated convex hulls, at a shrink factor of 0.5, for the outer boundary of blobs from the different classes that most closely match average feature values (Fig. 6A). Figure 6A presents representative 3D principal component analysis (PCA) views of node distribution and convex hulls for the most closely matching CAV1 WT and CSD mutant blobs to the average blobs features as well as overlaid X–Y 2D boundaries for the top ten matching blobs. Reduced size and altered shape are evident in 3D boundary convex hull representations and the 2D XY profiles suggesting that CSD mutant profiles are more compact and less variable than WT X–Y profiles. To quantitatively assess this, we analyzed volume and variance of convex hull volume at shrink factors of 0 (the most convex), 0.5, and 1 (the most indented) for the ten CAV1 WT and CSD mutant blobs from each cell that most closely matched the average feature values of all blobs from all the cells. Consistent with feature analysis of the point clouds (Fig. 5), all classes of CAV1 blobs were smaller for CSD mutant blobs than for WT blobs at all shrink values, and more significantly for S2 and S1B scaffolds (Fig. 6B).

Varying indentation of point clouds will not be reflected in average volume changes, but should be reflected in variance of convex hull volume amongst blobs, and more particularly with increasing convex hull shrink factors. As seen in Fig. 6B, analysis of variance of convex hull volume shows, as expected, that variance increases with increasing shrink factor and to a larger extent for WT blobs compared to CSD mutant blobs. Differences in convex hull variance between WT and CSD mutant blobs is not observed for caveolae blobs and is more pronounced for S2 and S1B scaffolds (Fig. 6B). This is consistent with the more pronounced differences in point cloud size and shape that we observed previously for S2 and S1B blobs (Fig. 5). This suggests that CAV1 distribution within non-caveolar CAV1 domains may be more variable than caveolae and that CSD mutation restricts the variable distribution of CAV1 within CAV1 blobs.

## Discussion

While SMLM imaging is now able to image proteins at nanometer scales, there is a challenge for analysis and quantification of SMLM to gain biological insight of proteins. Clustering methods for processing and quantifying the millions of localizations from SMLM data to obtain structural data and biological insight of proteins are relatively new and still being developed^50^. Application of machine learning and network-based cluster analysis to CAV1 previously identified caveolae and three distinct scaffold domains^8,10^. We now show that this novel structural analysis tool is able to detect structural changes to CAV1 oligomers induced by point mutations to the CSD.

To study expression of the CAV1 CSD mutant independently of endogenous CAV1, we generated a CAV1 knockout in the MDA-MB-231 cell line by adapting a CRISPR/Cas9 strategy to first sub-clone and create monoclonal populations of the parental cell line. The parental MDA-MB-231 cell population is highly heterogenous (Fig. 1) and we selected the F10 clone based on its elongated, mesenchymal morphology, CAV1- and Gal3-dependent migration and focal adhesion tension. Selection of the F10 clone over A10 was due to its higher CAV1 expression and increased CAV1-dependent cell migration, making it more suitable for study of CAV1 in metastatic breast cancer. The MC5 CAV1 knockout line showed decreased migration as previously reported when using CAV1 siRNA knockdown in MDA-MB-231 cells^45,51^. Further, we demonstrate that CAV1 knockout reduces focal adhesion tension that is rescued by CAV1 WT, but not by CAV1 CSD mutant. Together with previous results in PC3 cells that do not express CAVIN1 and lack caveolae^26^, these results in MDA-MB-231 cells confirm a role for CSD-dependent CAV1 regulation of vinculin tension independently of CAVIN1 expression. They further show that Gal3 is also a regulator of vinculin tension in MDA-MB-231 focal adhesions, consistent with previous results showing synergistic focal adhesion activation by Gal3 and CAV1 in other cancer cell lines^45–47,52^.

The CSD regulates CAV1 interactions and F92A and F92A/V94A CAV1 mutants disrupt CSD interactions with multiple proteins^22,23,25,28,29,32^. Electron microscopy analysis of the CSD single point mutation F92A in endothelial cells did not report any change in caveolae abundance at the cell surface^40^. Consistently, we see a minimal shift in the distribution of larger caveolae to smaller scaffolds, indicating that CSD integrity is not affecting the equilibrium of caveolae and scaffolds. This suggests that structural changes within caveolae or scaffolds are responsible for the functional differences of CSD mutant CAV1.

We show that CSD mutation reduces the size of CAV1 point clouds. This suggests that differences in the size of CAV1 point clouds reflect altered distribution of anti-CAV1 labeling and therefore structural differences in CAV1 distribution in caveolae and scaffolds. The decrease in node degree observed in caveolae, S2 and S1B structures upon CSD mutation may reflect a decrease in the relative abundance of detected CAV1 molecules. The network analysis method utilizes an iterative merge within 20 nm, which will merge CAV1 nodes closer than the 20 nm merge threshold. Together with the decreased blob size and volume, this suggests that there are either fewer CAV1 molecules within the CSD mutant blobs resulting in a smaller blob and decreased volume, or that the molecules are compacted beyond the 20 nm resolution of the system. This is supported by the reduced volume and increase density of CSD mutant CAV1 blobs. The lack of change in modularity of caveolae, S2 and S1B structures suggests that, despite a decrease in volume and nodes, large CAV1 structures are built from combinations of smaller S1A scaffolds^8^. CSD mutation may alter how S1A scaffolds combine to form larger structures. Greater shape changes due to the CSD mutation observed for S2 and S1B scaffolds compared to caveolae and S1A scaffolds suggest that recruitment of CAVIN-1 and formation of caveolae may reduce the impact of the CSD mutation on caveolae structure. CAV1-associated CAVIN1 is necessary for the formation of the polyhedral lattice coat on caveolae^14,18^ and deletion of the CSD prevents CAV1 induction of membrane curvature and caveolae biogenesis^41^. Differential effects of CAV1 CSD F92A/V94A mutation and CSD deletion on CAV1 structure and function, as well as the contribution of other caveolae associated proteins such as the membrane shaping protein PASCIN2 and EHD2^53,54^, require further analysis.

**Figure 7:**
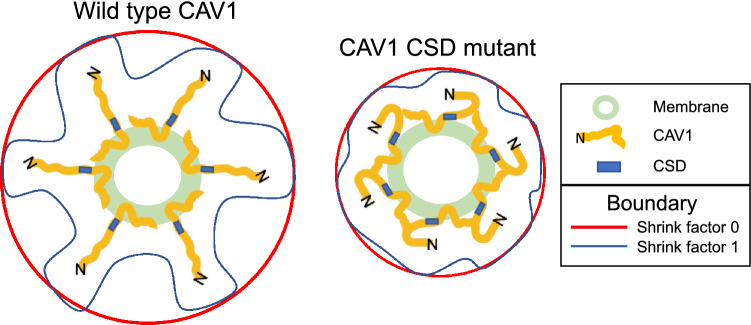
Schematic diagram of convex hull analysis of CAV1 WT and CSD mutant scaffold blobs. Increased variance of the convex hull boundary at high shrink factors (most indented) compared to low shrink factors (the most convex) is suggestive of the more variable distribution of N-terminal CAV1 labeling in CAV1 WT relative to CAV1 CSD mutant scaffold domains. CSD indicated by blue box and N-terminus of CAV1 by N. Membrane is in green and convex hull boundary of CAV1 point clouds at shrink factor 1 in blue and at shrink factor 0 in red. Representative CAV1 domains are shown containing 6 CAV1′s and do not correspond in size, number or scale to any known CAV1 scaffold. Adapted from^39^.

The theorized location of the CSD either embedded or in close proximity to the cell membrane has led to debate as to whether the CSD is accessible to interact with other proteins and what the impact of the CSD has on CAV1 structures^27,36,37^. To better understand the decreased size of the CSD mutant blobs, we visualized representative CAV1 WT and CSD mutant point clouds as convex hulls connecting nodes on the outer surface of the point cloud. Convex hull analysis at increasing shrink values detects indentation of the outer surface of CAV1 point clouds. Point cloud indentations are not attributable to localization errors or multiple blinking of fluorophores since sample preparation, fluorophores and image acquisition was the same between CAV1 WT and CSD mutant and identical merging and preprocessing were applied across classes. By EM, the caveolae membrane is smooth and does not show any evident indentations, even for the CAV1 CSD mutant^40^. The anti-CAV1 antibody (rabbit anti-caveolin-1; sc-894) used targets the CAV1 N-terminus region and indentations of the convex hull of the CAV1 point cloud may therefore report on differential extension of CAV1 N-terminus labeling away from the caveolae membrane (Fig. 7). Supporting a role of the N-terminal CAV1 region in the larger structure of WT caveolae and scaffolds, in vitro cryo-EM of extracted CAV1 8S complexes showed that truncation of the first 31 amino acids of the N-terminus results in smaller 8S structures^55^. Our data suggest that the CSD mutation shifts the N-terminus inwards towards the membrane, potentially reducing accessibility of the CSD to interact with effector proteins^27,36,37^. Increased variance of CAV1 WT blob volume is suggestive of extension of the CAV1 molecule and therefore CAV1 labeling away from the blob, potentially exposing the CSD and enabling CSD protein–protein interactions. The larger structural changes to CAV1 blobs upon CSD mutation for S2 and S1B scaffolds, including variance of convex hull volume, supports a role for these CAV1 domain classes in CSD-mediated interactions.

The CSD mutation impacts the structure of both caveolae and scaffolds. This suggests that CSD function may be associated with both caveolar and non-caveolar CAV1 domains. However, CSD dependence of pCAV1 focal adhesion tension and migration was observed in PC3 prostate cancer cells that lack CAVIN1 and caveolae, indicating that the CSD can act exclusively through scaffolds^26^. How the CSD-dependent structural changes to both caveolae and scaffolds identified here by SMLM network analysis contribute to the CSD-mediated interaction of CAV1 with various signaling proteins and to the role of the CSD in disease progression^56^ remains to be determined.

## Materials and methods

### Antibodies, plasmids and siRNA

Primary rabbit anti-CAV1 antibody (sc-894), rabbit anti-Gal3 antibody (sc-20157) were purchased from Santa Cruz, rabbit anti-p-CAV1 (#3251) from Cell Signaling, anti-β-actin (A2228) from Sigma-Aldrich, mouse anti-GM130 (610822) from Biosciences and mouse anti-KDEL (ab12223) from Abcam.

Testing of CAV1 antibodies with the CAV1 KO MDA-MB-231 cell line found that Santa Cruz SC-894, used in this paper and now discontinued, and Cell Signalling antibody 3267 were highly specific. Secondary goat anti-rabbit Alexa647 (A-21245), goat anti-rabbit Alexa488 (A32731), and goat anti-mouse Alexa568 (A11031) were purchased from Thermo Fisher Scientific. Horseradish peroxidase (HRP)–conjugated mouse and rabbit secondary antibodies were purchased from Jackson ImmunoResearch Laboratories. Fluorescent labelling F-actin using Alexa Fluor 488-Phallodin (A-22287) was purchased from Thermo Fisher Scientific.

In order to generated Myc tagged human wildtype CAV1 or carrying CSD mutation (F92A/V94A), CAV1-myc-mRFP wt or CSD mutant were used as template DNA and PCR (Q5, Qiagen) amplified using the following sets of forward and reverse primers (5′GGA AGC TTA GCA TGT CTG GGG GCA AAT AC3′; 5′GGG ATC CTC ACA GAT CCT CTT CTG AGA TGA G3′). The PCR products were TA cloned and sequence verified for fusion of the Myc tag, released using EcoR1 and re-cloned into pCDNA3 at EcoR1 site. Positive clones were sequence verified for the correct orientation and used for transfection.

CAV1, Gal3 and control siRNA were purchased from Dharmacon (human siCAV1: L-003467–00; custom siGal3^57^; siControls: D-001210–01). VinculinTS was a gift from Martin Schwartz (Addgene plasmid # 26019; http://n2t.net/addgene:26019; RRID: Addgene_26019).

### Cell culture, transfection, western blot

MDA-MB-231 cells (cell line validated by single tandem repeat analysis at the Centre for Applied Genomics (SickKids, Toronto, Canada) were cultured in RPMI-1640 medium (Thermo-Fisher Scientific Inc.) supplemented with 10% fetal bovine serum (FBS, Thermo-Fisher Scientific Inc.) and 2 mM L-Glutamine (Thermo-Fisher Scientific Inc.). All cells were tested for mycoplasma using a PCR kit (Catalogue# G238; Applied Biomaterial, Vancouver, BC, Canada)^51^. Plasmid transfection or small interfering RNA (siRNA) transfection was done 24 h after plating of the cells, using Lipofectamine 2000 (Life Technologies, Thermo Fisher Scientific) following the manufacturer’s protocol^26,51^. Lipofectamine 2000 transfection of the CAV1 WT and CSD mutant plasmids had a transfection efficiency of approximately 30%.

For Western blot, cells were pelleted and then washed with cold phosphate-buffered saline (PBS) and resuspended in lysis buffer (20 mM Tris–HCl, pH 7.6, 0.5% NP-40, 250 mM NaCl, 3 mM EDTA, and 3 mM ethylene glycol tetraacetic acid containing Roche complete protease inhibitor cocktail and PhosSTOP phosphatase inhibitor) for 30 min at 4 °C. The sample was centrifuged at 13,000 rpm at 4 °C, and the supernatant was stored at − 80 °C. Normalized concentrations of proteins were separated on a 12% SDS–PAGE, electroblotted onto nitrocellulose (GE Healthcare Life Sciences), probed with indicated antibodies and HRP-conjugated secondary antibodies. Detection was performed using enhanced chemiluminescence^26^.

### Subcloning and CRISPR/CAS9 knockout

CAV1 knockout MDA-MB-231 cell line was generated using GeneArt-CRISPR/Cas9 Nuclease vector containing OFP (Orange Fluorescence Protein, A21174, Life Technologies, USA), as previously described^58^. Guided RNAs (gRNAs) were designed using http://crispr.mit.edu and off-targets checked using http://www.rgenome.net/cas-offinder/ (RGEN tools). Forward and reverse oligonucleotides (5′CCA CGG GCC AGC ATG TCT GTTTT-3′ and 3′-GTGGC GGT GCC CGG TCG TAC AGA-5′) were in vitro annealed to generate gRNA duplex, which was cloned into the GeneArt linear vector as suggested in supplied manual. The sequence verified GeneArt plasmid containing CAV1 specific gRNA duplex was transfected 24 h after plating the F10 sub clone of MDA-MB-231 cells using Lipofectamine 2000 (Life Technologies, Thermo Fisher Scientific). 36 h post incubation, genomic DNA was extracted from the transfected cells to perform GeneArt Genomic Cleavage Detection assay (A24372, Invitrogen, USA) to check the cleavage efficiency. Based on optimized cleavage efficiency, transfection was repeated and post 36 h incubation, OFP expressing cells were FACS sorted and cloned by limiting dilution in 96 well plates. Single colonies were replicated, expanded in 12 well plates; one set was frozen and stored in liquid N2 whereas the other set was subjected to lysate preparation, SDS-PAGE and CAV1 western blot analysis. Select clones were expanded, tested for mycoplasma and stored as multiple freeze-downs. Genomic DNA was extracted from the clone showing CAV1 knockout by western blot, and used for PCR amplification of approximate 800 bp fragment flanking Exon 1 of CAV1 using Q5 polymerase (Qiagen, USA). Products were TA cloned and sequenced to verify INDEL at the initiation codon of the gene.

### F-actin labeling and confocal microscopy

Cell morphology was assessed by Alexa488 phalloidin (Life Technologies, Thermo Fisher Scientific) labeling of clones fixed with 4% paraformaldehyde (PFA; 15 min), permeabilized with 0.1% Triton X-100 (10 min) and then blocked with 2% BSA, in PBS containing 1 mM MgCl_2_ and 0.1 mM CaCl_2_ (PBS-CM). Coverslips were mounted with Prolong Gold (Life Technologies, Thermo Fisher Scientific), and images were acquired with a 63X/1.4 oil objective on a Leica TCS SP5 confocal microscope^26^.

### Transwell migration

Cells were trypsinized, counted, resuspended in medium containing 0.5% serum and then transferred to 8-μm cell culture inserts (BD Falcon) placed into 24-well plates containing complete medium. After 16 h, cells were removed from the top of the filter using a cotton swab, and migrating cells on the bottom of the filter were fixed with 4% PFA and stained with 5% crystal violet^26^.

### FRET

FRET was performed on a Leica TCS SP8 confocal microscope using a 63 × water immersion objective as described previous^26^. Cells were fixed with 4% PFA 24 h after transfection and then labelled with anti-CAV1 primary and Alexa Fluor 647–conjugated secondary. Using the FRET acceptor bleaching Leica software module, cells expressing both the VinculinTS and respective CAV1 were chosen and regions of interests were drawn around visible focal adhesions and excited with the 515-nm laser line to bleach the Venus channel. FRET efficiency was calculated by the software based on the intensity in the prebleach image and postbleach image of the mTFP and Venus channels.

### SMLM imaging

Coverslips (NO. 1.5 H) were sonicated 1 h with 1 M aqueous potassium hydroxide, followed by sonication in ethanol 1 h and then washed with Mili-Q water. Cells were plated on coverslips coated with fibronectin (Sigma-Aldrich Inc.) for 24 h before fixation. Cells were fixed with 4% PFA for 15 min at room temperature, rinsed with PBS-CM, permeabilized with 0.2% Triton X-100 in PBS/CM, incubated with Image-iT FX Signal Enhancer (Thermo Fisher Scientific) and blocked using BlockAid Blocking Solution (Thermo Fisher Scientific). Then the cells were incubated with the primary antibody for 12 h at 4 °C and with the secondary antibody (Alexa Fluor 647-conjugated goat anti-rabbit; Thermo-Fisher Scientific Inc.) for 1 h at room temperature. The antibodies were diluted in SSC (saline sodium citrate) buffer containing 1% BSA, 2% goat serum and 0.05% Triton X-100. Cells were washed with SSC buffer containing 0.05% Triton X-100 and post-fixed with 3% PFA for 15 min followed by washing with PBS/CM. 0.1 µm TetraSpeck Fluorescent Microspheres (Thermo Fisher Scientific) were added to the sample as fiducial markers. Before imaging, the imaging buffer was freshly prepared with 10% glucose (Sigma-Aldrich Inc.), 0.5 mg/ml glucose oxidase (Sigma-Aldrich Inc.), 40 μg/mL catalase (Sigma-Aldrich Inc.), 50 mM Tris, 10 mM NaCl and 50 mM β-mercaptoethanol (βME; Sigma-Aldrich Inc.) in Milli-Q water.

The slides were mounted and sealed on a glass depression slide. GSD super-resolution imaging was performed on a Leica SR GSD 3D system using a 160 × objective lens (HC PL APO 160 × /1.43, oil immersion), a 642 nm laser line and a EMCCD camera (iXon Ultra, Andor). Epi-illumination was applied at full laser power for the pumping process to bring the fluorophores into the dark state, while a TIRF illumination with 100-nm penetration depth was applied for acquisition. Acquisition was done for 10 min with camera exposure time at 10 ms/frame to generate the event list. Representative super-resolution images were generated using the Leica SR GSD software with XY pixel size 20 nm, Z pixel size 25 nm and Z acquisition range ± 400 nm. The event list was processed used fiducial markers to correct for drift^8,10^.

### Cluster analysis

3D SMLM Network Analysis pipeline^10^, consisting of computational modules to pre-process and post-process the 3D SMLM data, was leveraged to analyze the SMLM CAV1 data. We used the iterative merging algorithm at 20 nm merge threshold to correct for multiple blinking of a single fluorophore artifact. To remove background and monomeric localizations, we used a filtering algorithm based on per-node graph/network features for the corrected localizations. We construct an unweighted network/graph and extract the node degree for every individual localization and compare it with the node degree of a random graph, retaining nodes with features that are different from the random graph.

The mean-shift algorithm^59^ is applied to the filtered 3D localizations to segment the 3D point clouds into clusters/blobs. After that, a set of 28 blob-wise features (Table S1) are extracted to characterize distribution, anisotropy, distance to centroid, node degree and other network features and their corresponding statistical features^10^. The 28-features are then used to identify the blob group based on the X-means algorithm^60^ that finds the optimal number of clusters/groups in the underlying 28-feature space using the Bayesian information criterion.

### Principal component analysis

Usually, PCA is used for dimensionality reduction and feature selection, outlier detection, trend observation, and to uncover relationships between features, etc. in the data. Here, we used PCA to map/project the blobs into space where the direction of the localization dispersion (Eigenvectors) is unified. The blobs appear in various orientations (XYZ) in the original cell space. We used PCA to map the blobs into a domain such that PC1 represent the dimension of the highest variance of the distribution of the blob’s localizations, PC2 is the dimension of the second highest variance, and PC3 is the dimension of the lowest variance for the localizations. PCA helps us to qualitatively compare blobs of the same group/class before and after the mutation to show the size/volume and shape changes that happened to the blobs. Hence, we use PCA to visually show and overlay the 3D localizations of the blobs before and after mutation. Using PCA, we show the correspondence between our qualitative and quantitative analysis of the Cav1 blobs in both populations/datasets. For example, PCA helps us to show the variations of the feature across the blobs from the two populations where we can show that the volume and shape of the blobs are varied before and after the mutation as depicted in (Fig. 6). In addition, we extracted the 2D XY boundaries (at various shrinking factors) to visualize the size and shape of blobs in the plane of the membrane.

### Blob/cluster boundary analysis

Given a 2D or 3D point cloud P (where P = {(x,y)} or {(x,y,z)}) representing the localizations of a blob, a blob boundary B (where B ⊆ P) can be formed from a set of points that, when connected together as a boundary in 2D or a triangle mesh in 3D, envelops all points P. In our implementation, we used MATLAB’s *boundary* function that is parameterized by a *shrink factor* scalar parameter between 0 and 1 to controls the tightness of the boundary in 2D or the triangulation in 3D. Setting the factor to 1 produces the convex hull of the points, while a value of 0 produces the tightest single-region boundary.

## Supplementary Information

Supplementary Information.
