# Supplementary material for: Single molecule network analysis identifies structural changes to caveolae and scaffolds due to mutation of the caveolin-1 scaffolding domain
Source: Sci Rep. 2021 Apr 8;11:7810. doi: 10.1038/s41598-021-86770-6 (PMC8032680; doi:10.1038/s41598-021-86770-6)
Supplement: Supplementary file 1 — Supplementary Information. [file 41598_2021_86770_MOESM1_ESM.pdf]

Single molecule network analysis identifies structural changes to caveolae and scaffolds due to mutation of the caveolin-1 scaffolding domain

Timothy H. Wong[1*], Ismail M. Khater[2*], Bharat Joshi[1], Mona Shahsavari[2], Ghassan Hamarneh[2^#,] Ivan Robert Nabi[1,3^#]

SUPPLEMENTARY FIGURES

Supplemental Fig 1. Western blot of the endogenous CAV1, Gal3 and β-actin in MDA-MB-231 sub-clones after siRNA knockdown of CAV1 and Gal3. See Supp. Fig. 3 for complete blots.

Supplemental Fig 2. Confocal images of CAV1 KO MDA-MB-231 cells transfected with CAV1 WT or CSD mutant and labeled for either Golgi GM130 (A) or endoplasmic reticulum anti-KDEL antibodies (B).. CAV1 labeling of the constructs is shown in green and Golgi or endoplasmic reticulum labeling in red. Peripheral CAV1 puncta corresponding to cell surface CAV1 are shown in insets. Scale bar, 10 µm; insets, 2 µm.

Supplemental Fig 3. Overall changes in all 28 features for all blobs. Quantification of blob localizations distribution, anisotropy, distance to centroid, clustering coefficient, and blob network and node degree features between MC5 cells transfected with CAV1 WT or CSD mutant (two-tailed unpaired t test; *p < 0.05; **p < 0.01; ***p < 0.001; ****p < 0.0001).

Supplemental Fig 4. Silhouette criterion plots of the X-means clustering results for determining the four blob groups in CAV1 wildtype and the CSD mutant transfected cells from experiment 4. The colours correspond with the following groups: Green are S2 scaffolds, blue are caveolae, red are S1A scaffolds and purple are S1B scaffolds.

Supplemental Fig 5. Complete Western blots for Figure 1A (A), Figure 1D (B) and Supp. Fig. 1 (C).

Supplemental Table 1. Listing and description of the 28 blob features. This table is a slightly modified version of Supplemental Table S2 in a previous publication [10].

SUPPLEMENTARY TABLE

Supplemental Table 1. Listing and description of the 28 blob features. This table is a slightly modified version of Supplemental Table S2 in a previous publication [10].

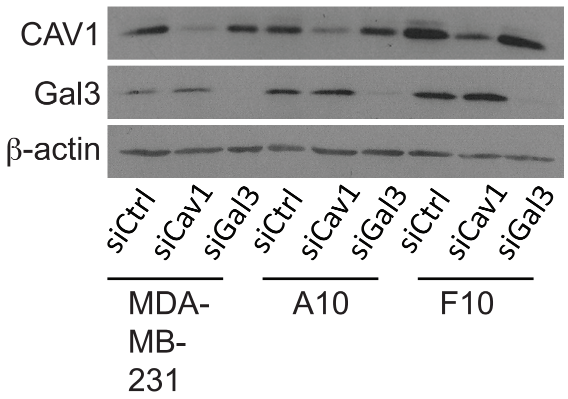

Supplemental Figure 1

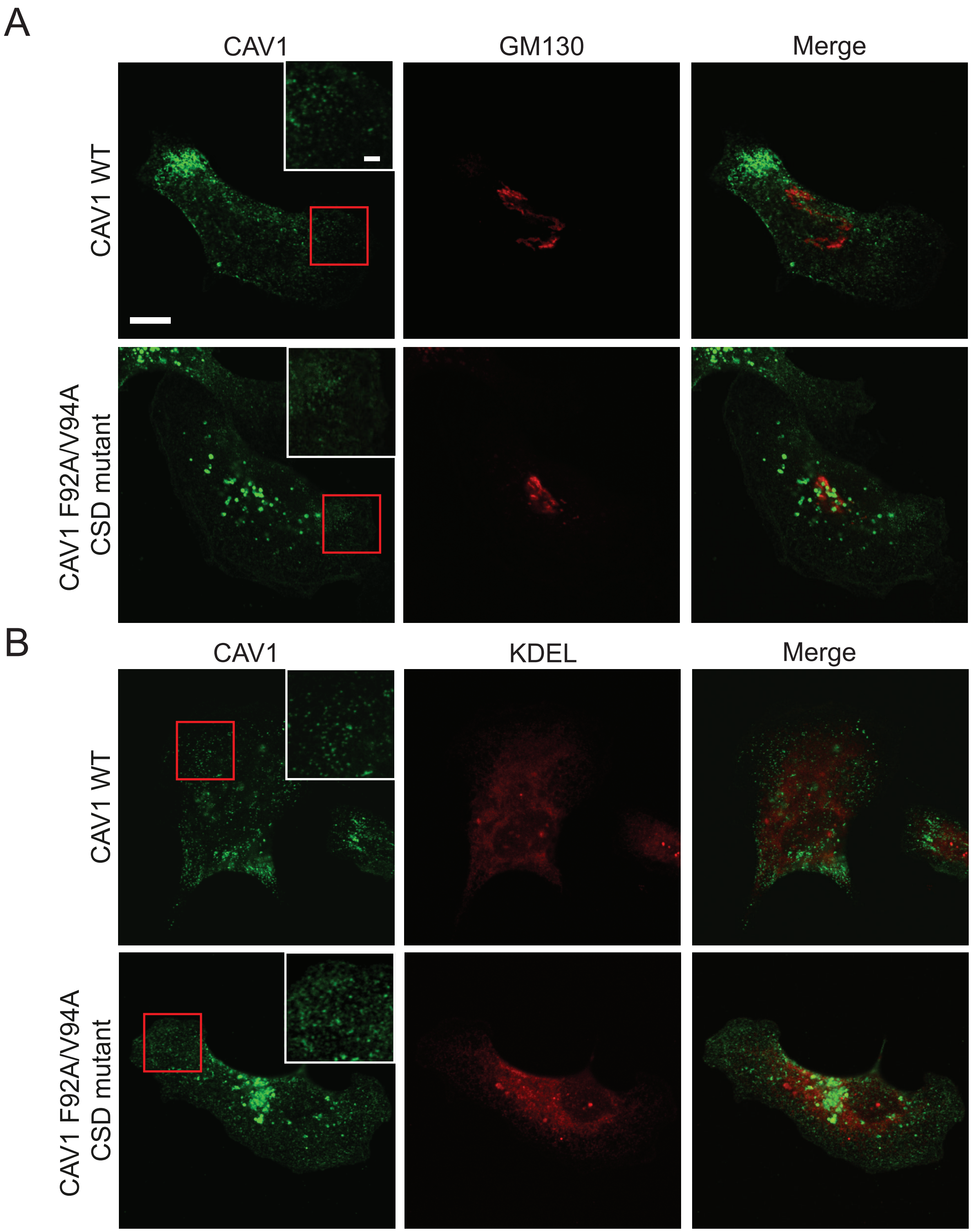

Supplemental Figure 2

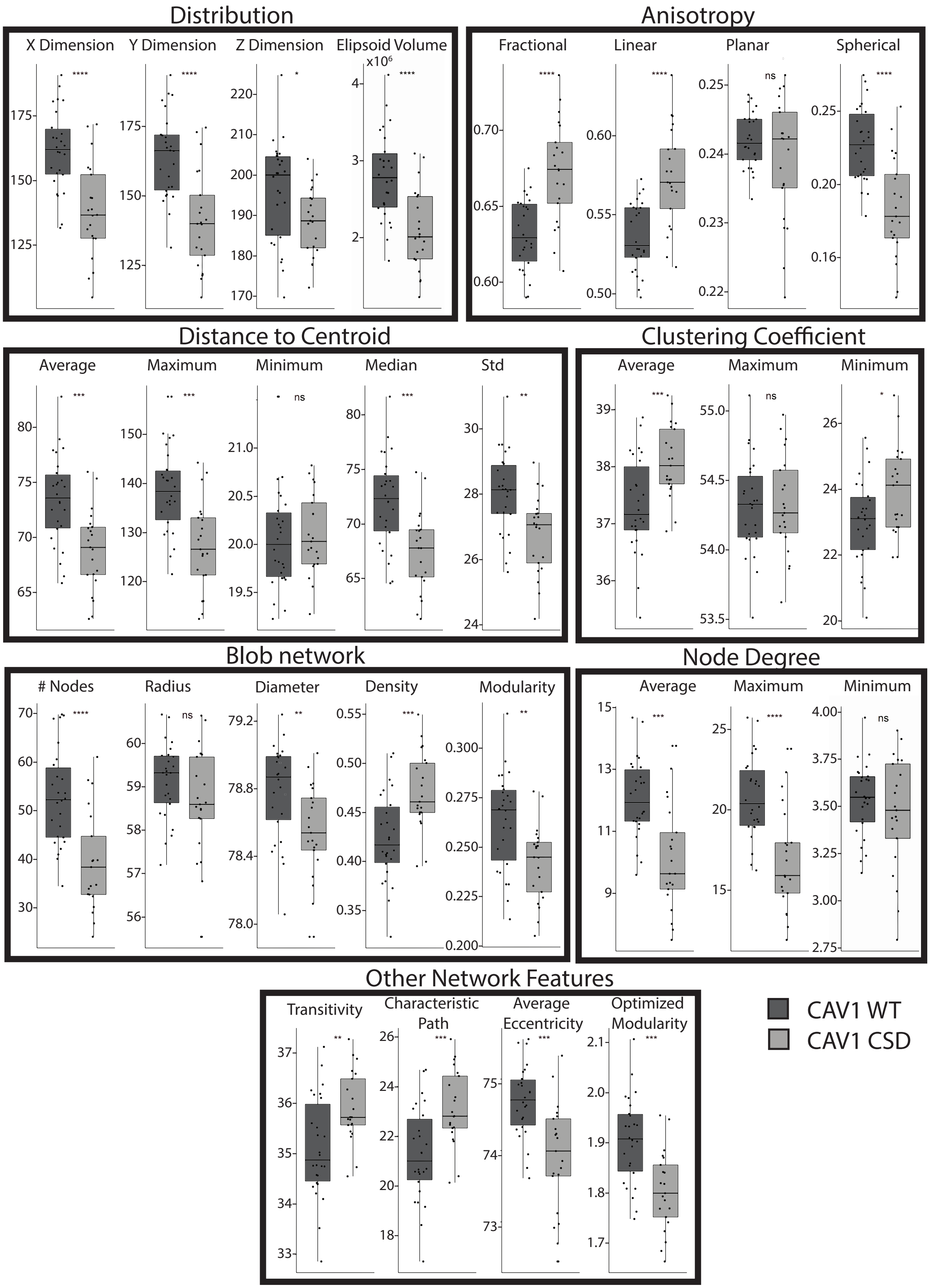

Supplemental Figure 3

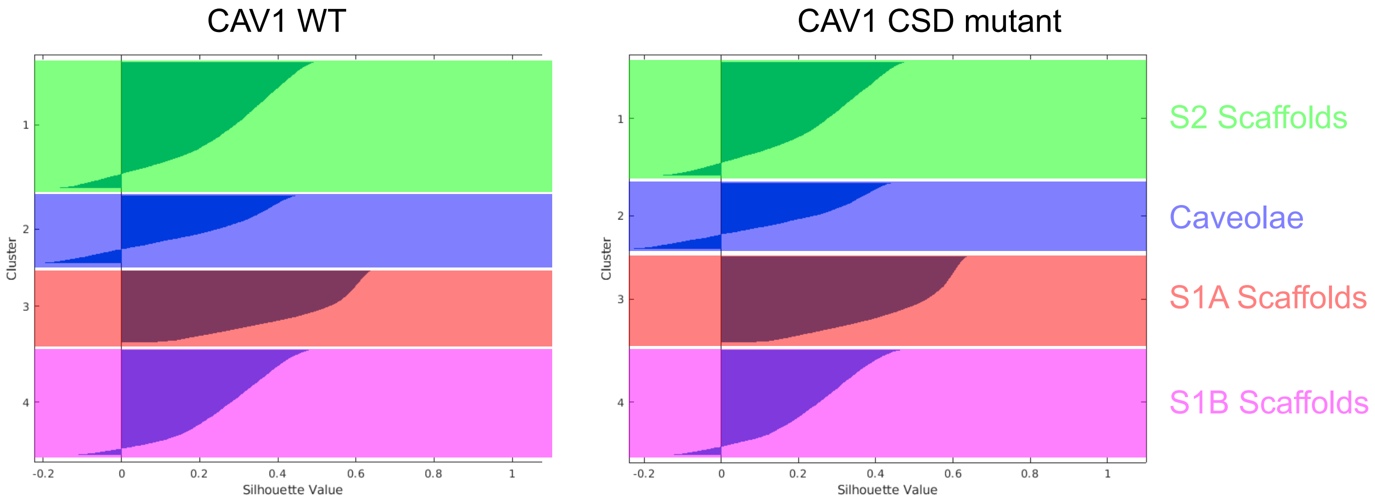

Supplemental Figure 4

Supplemental Figure 5A. Complete Western blots for Figure 1A
(portions of blots used in red boxes; please note that the CAV1-Gal3-β-actin and pCAV1/β-actin blots were from different experiments)

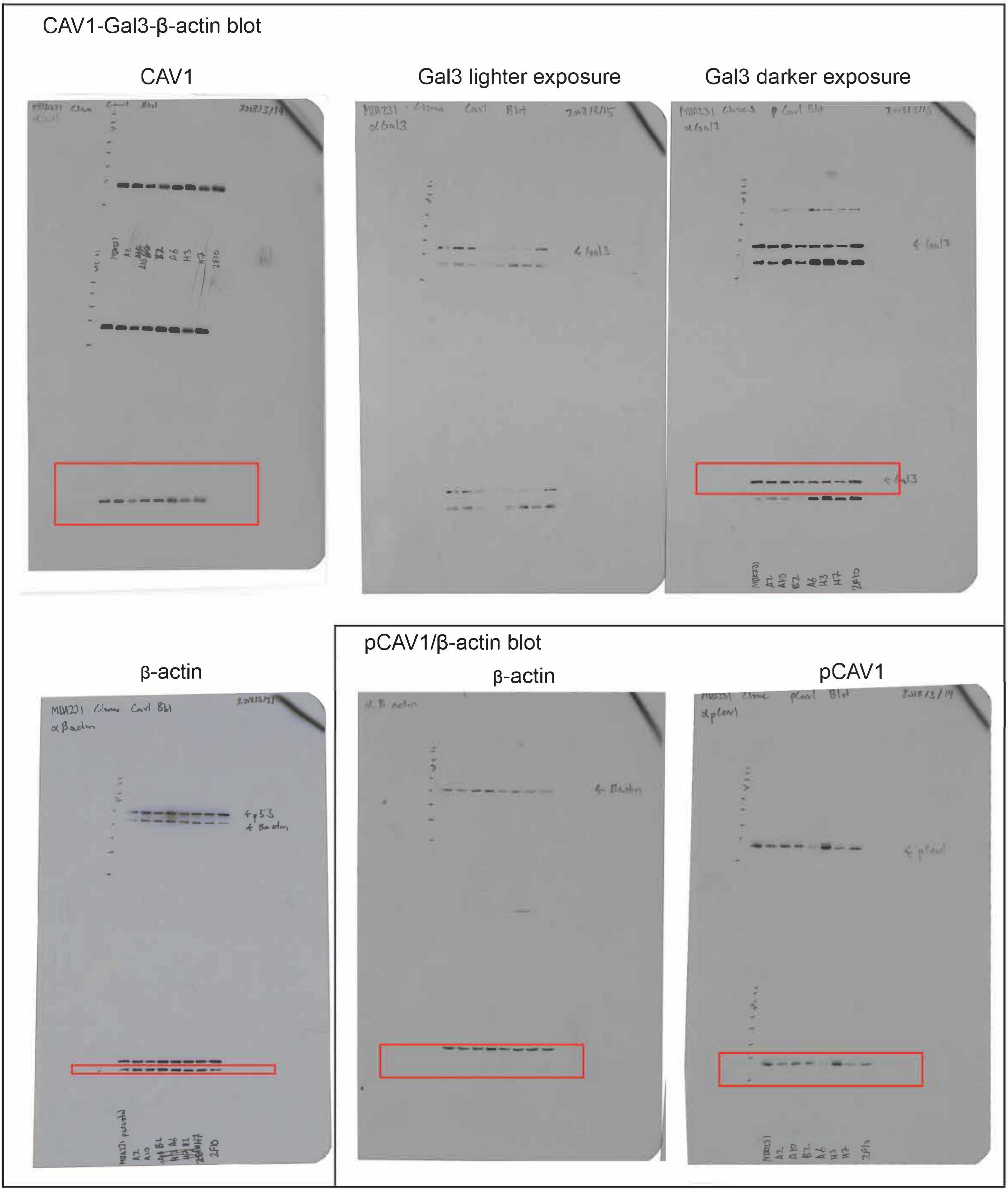

β-actin

Supplemental Figure 5B. Complete Western blots for Figure 1D (portions of blots used in red boxes)

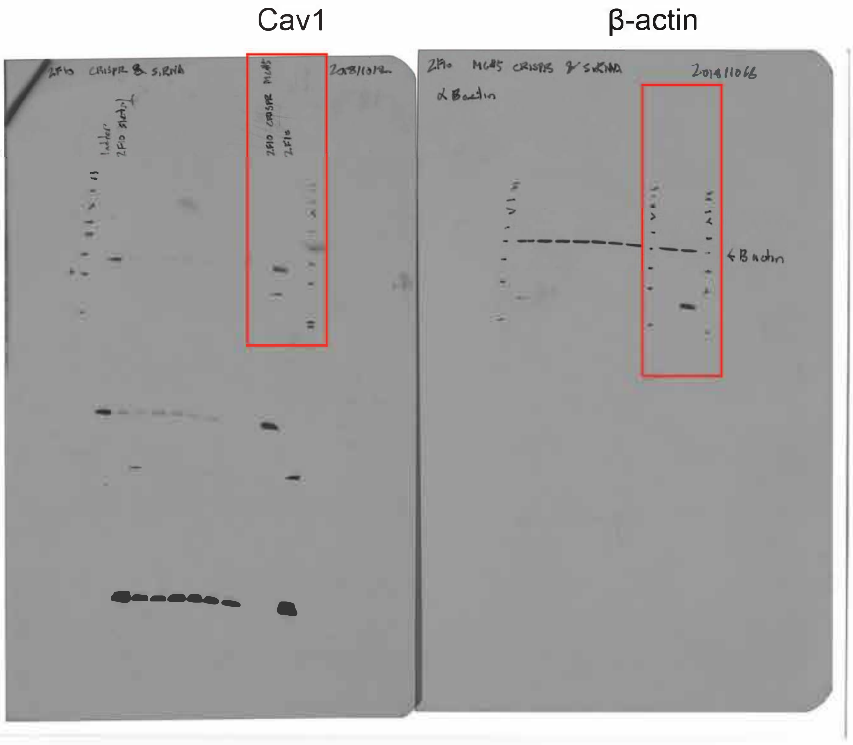

Supplemental Figure 5C. Complete Western blots for Supplemental Figure 1 (portions of blots used in red boxes)

CAV1

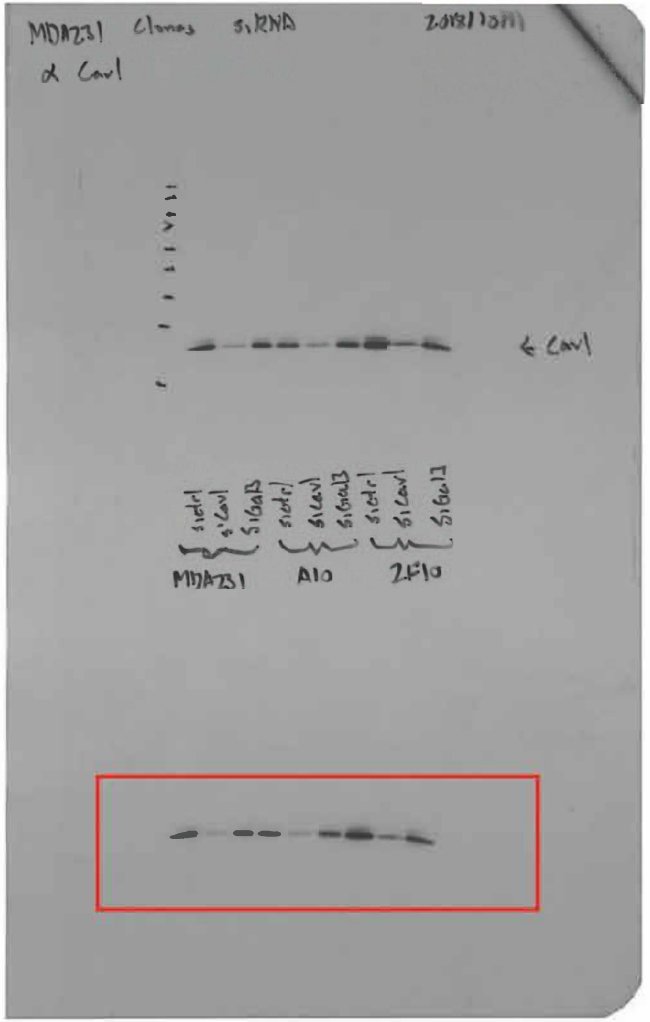

β-actin

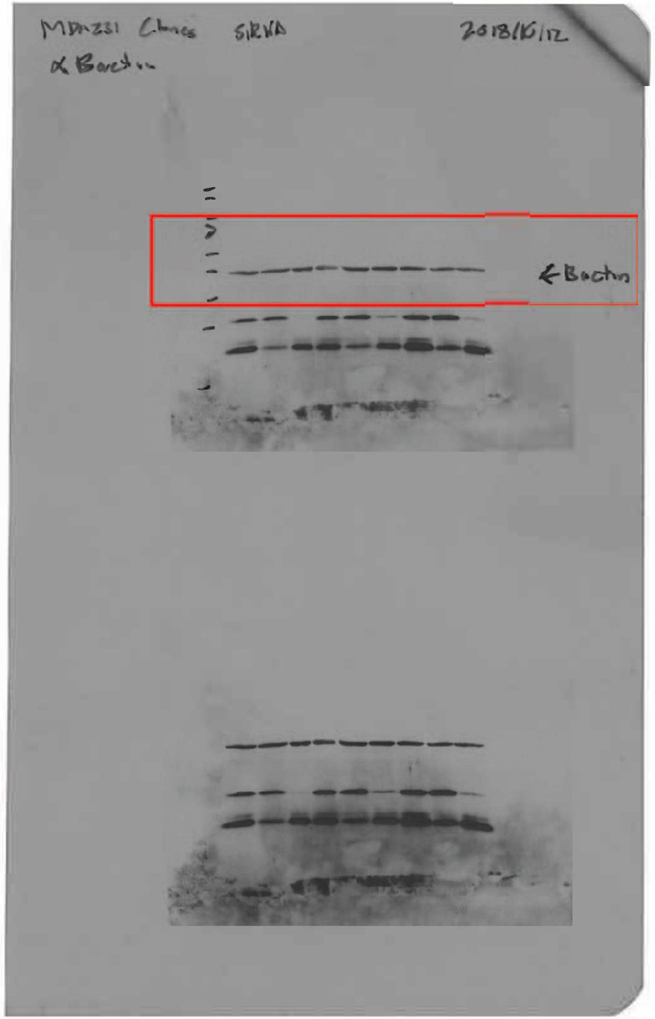

Gal3

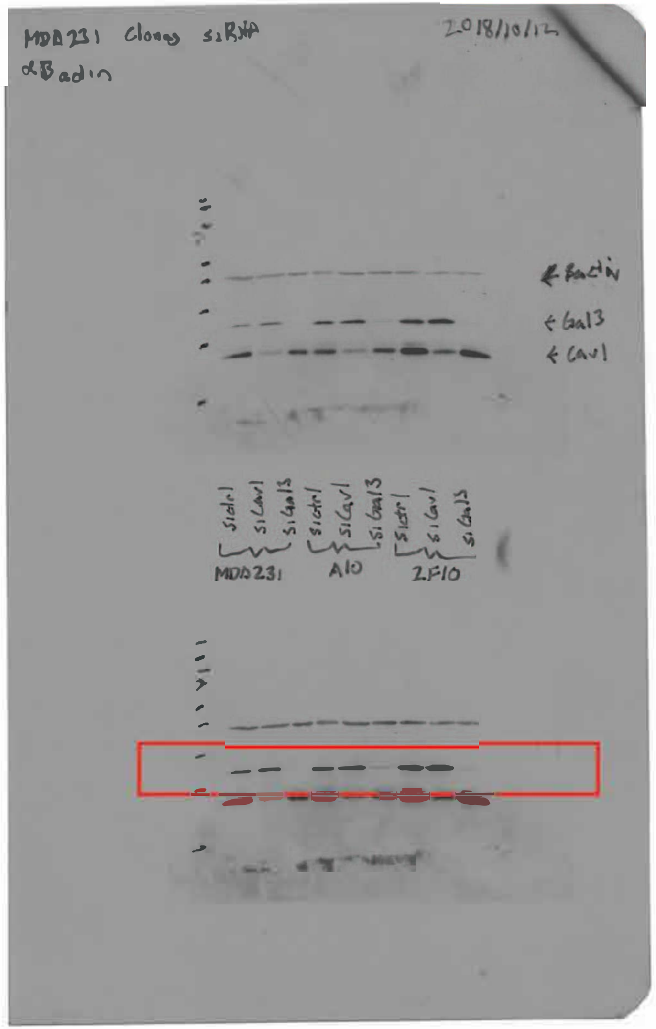

Supplemental Table 1. Listing and description of the 28 blob features.

Feature	Description
Distribution	
X Dimension	Distribution of the point cloud along the X-dimension
Y Dimension	Distribution of the point cloud along the Y-dimension
Z Dimension	Distribution of the point cloud along the Z-dimension
Elipsoid Volume	Ellipsoid volume of the 3D point cloud of the blob
Anisotropy	
FA	Fractional anisotropy
CL	Linear anisotropy
CP	Planar anisotropy
CS	Spherical anisotropy
Distance to Centroid	
Average	Average distances of the nodes to their centroid
Maximum	Maximum distance of the nodes from their centroid
Minimum	Minimum distance of the nodes from their centroid
Median	Median distance of the nodes to their centroid
STD	Standard deviation of the nodes distances to their centroid
Clustering Coefficient	
Avgerage	Average clustering coefficient value of the nodes
Maximum	Maximum clustering coefficient value of the nodes
Minimum	Minimum clustering coefficient value of the nodes
Blob Network	
# Nodes	Number of nodes
Radius	Blob’s network/graph radius
Diameter	Blob’s network/graph diameter
Density	Blob’s network/graph density
Modularity	Blob’s network/graph modularity measure
Node Degree	
Avg. degree	Average node degree within the blob
Max. degree	Maximum node degree within the blob
Min. degree	Minimum node degree within the blob
Other Network features	
Transitivity	Transitivity measure of the nodes
Characteristic path	Characteristic path of the nodes
Average eccentricity	Average eccentricity measure
Optimized Modularity	Average optimized modularity for the blob’s network
